# Rheumatism and Gout

**Published:** 1903-09-26

**Authors:** 


### RHEUMATISM AND GOUT.

Woods Hutchinson[1] ("The Meaning of Uric Acid and the Urates") discards the older theories that these bodies are due to imperfect combustion of nitrogenous food or a reversion to a more primitive form of excretion. He holds that the appearance of uric acid and urates in the excretions is due to the destruction of leucocytes in their battle with toxins, and that the coincident increase in phosphate excretion is due to the fact that leucocyte nucleins are a combination of phosphoric acid and purins. He shows that the toxic and irritative properties of uric acid have been over-estimated and cannot cause the symptoms of gout. Leucocytosis goes with increased elimination of purins, and this suggests a division of all persons into three classes: (1) *Acytic*, in whom leucocytosis (as an index of resisting power) is deficient; these will suffer from acute infections such as tuberculosis, typhoid and rheumatic fevers. (2) *Hemicytic*, in whom the defensive power of the leucocytes is moderate but inadequate; these will suffer from gout because the leucocytes, unable to completely destroy the toxins, will die in large numbers and produce uric acid excretions. (3) *Hypercytic*, in whom the leucocyte power is quite efficient; these will be protected from ordinary intoxications. The classes he admits are liable to incidental modifications. Regarding gout, therefore, as due to the entry of a poison into the body, either from without (such as lead, and certain carbohydrates and ethers) or from within (auto-intoxication from the intestine), the treatment will be not to limit nitrogenous foods, which cannot affect either the cause or symptoms of the disease (the increase in uric acid being from "endogenous" sources alone), but to limit intestinal putrefaction, to increase general elimination, and to regulate the habits of the patient, so as if possible to raise him from the hemicytic to the hypercytic class. Williamson[2] has shown that in 11 cases, mostly cases of phthisis, increased excretion of phosphoric and uric acids corresponded to a decrease in the older forms of leucocytes but not in the younger cells, and *vice versâ*. This was more evident in children than adults. This he took as conclusive proof that the leucocyte destruction is the cause of the increased uric acid excretion. Halliburton thought that considering their total volume in the body, too much stress was laid on leucocytes as a cause of increased purin excretion. Hall[3] has estimated the amount of purin bodies in various foodstuffs. Glands contain most purin; then come red and white meats, fish, and chicken, with about the same quantity in each; of vegetables, peas, beans, and lentils are richest. Bread, eggs, rice, tapioca, green vegetables, and wines contain no purin. Milk and its derivatives may contain a trace. Purin bodies in beer are due to "yeasting." In a series of experiments on men he found that about half the exogenous meat-purins are excreted in 48 hours. The same is apparently true of vegetable and beer purins. The remaining half, after undergoing further oxidation (mainly in the liver), is excreted as urea. Endogenous purins are supposed to be excreted in the same proportion. The writer does not think that leucocyte destruction has much effect on the amount of endogenous purins. Bain,[4] as the result of observations on three patients, states that (in the order given) sidonal, lysidin, piperazin, and piperidin increase the excretion of uric acid in gout; that lithium benzoate and urotropin have no effect upon it; and that colchi-sal diminishes it while increasing that of the conjugated sulphates. Tetra-ethyl ammonium sulphate is a solvent merely on account of its alkalinity. Good[5] gives a history of the medicinal use of lithium salts. Their solvent properties on uric acid only occur in very strong solutions, and they are practically not absorbed by the skin. By means of over 30 experiments on animals in which lithium salts were given in various ways, he shows that they are mainly excreted in the urine, but where vomiting and diarrhœa occur more is excreted into the alimentary canal. In all his cases, sooner or later fatal gastro-enteritis occurred. Lithium is not a diuretic, and the sedative action of its bromide is due to the bromide ion. Fifteen to 20 grains of lithium carbonate have caused gastro-enteritis in man.

Walker and Beaton[6] have isolated from 15 cases of acute and subacute rheumatism, chorea, and endocarditis a micrococcus which they consider identical with that of Triboulet, Wassermann, Meyer, Paine and Poynton, and others. It has no distinguishing cultural features, but in two trials gave Marmorek's test of specificity, viz., it grew well in filtered cultures of streptococci. It produces an acid (*not* lactic) in alkaline broth; injected into animals it causes arthritis, pericardial and pleural effusions, and vegetations on the cardiac valves; in sufficient doses it kills rabbits in 48 hours; and can be re-cultivated from the lesions produced. True chorea was never seen, even in quite young animals. Stengel[7] does not think that the microbic origin of rheumatic fever has been conclusively shown, and is inclined to regard it as a symptom-complex (as is pneumonia) which may be caused by various micro-organisms. He traces the connection between chronic rheumatism and rheumatic fever, and draws a sharp distinction between these and the arthritis occurring in certain nervous diseases which should not be termed rheumatic. While not supporting the "nervous" theory of rheumatic fever, he considers chronic osteo-arthritis a degenerative process depending on *primary* nerve-lesions. To this condition the disease characterised by "Heberden's nodes" is closely allied. Dreschfeld[8] has pointed out that the soil is at least as important as the seed in the production of acute rheumatism, and that moreover considerable doubt still exists as to the identity of the micro-organism described. He considers septic and pyæmic arthritis closely allied to acute rheumatism. Salicylates are merely useful as antagonising symptoms. The occasional occurrence of an acute osteo-arthritis and the arthritis observed in fevers are notable. The latter might be due to (1) toxins, (2) micro-organisms, (3) a mixed infection, (4) true rheumatic fever complicating the disease. Chronic rheumatic and chronic osteo-arthritis are hardly distinguishable clinically. Under the latter heading a group of diseases is included wherein cases showing Heberden's nodes should not always be placed—some of these being gouty. Subsequently Dixon Mann threw doubts on the specificity of Menzer's micrococcus, and Wilkinson said that salicylates probably only neutralised toxins, and agreed with Dreschfeld in advising moderate doses. Professor Wild drew attention to the similarity between the evanescent arthritis in rheumatism and the fugitive rash in urticaria. Malim[9] has described a case of rheumatism alternating with asthma, and another in which some symptoms of Graves' disease were present. Paul[10] has noted an arthritis in Ceylon following non-amœbic dysentery, which, but for the escape of ligaments and fasciæ, resembles gonorrhœal arthritis. Forster[11] has recorded a case of a child in which tonsillitis, acute rheumatism, chorea, iritis, and endocarditis followed each other without intermission. The child recovered after about six months' illness. At the Cairo Congress in December, 1902, Professor Bouchard[12] recommended the subcutaneous injection of from $\frac{1}{2}$ to 3 grains of sodium salicylate in pleurisy, pericarditis, and near the site of the joint lesions in acute rheumatism. He had, however, failed to influence endocarditis by this method. Walsh[13] recommends large doses of salicylates in rheumatic fever (90 to 120 grains daily). Alkalies should be combined with them to prevent heart complications. Nervous and digestive disturbances, and "salicylic dyspnœa" are contra-indications. O'Conor[14] has reported fully a further series of 20 cases of acute rheumatism in which ordinary treatment having failed, the joints were opened aseptically and drained with happy results in all instances. Details of the operative technique are given.

**Angeio-neurotic Œdema.**—Griffith[15] reports at length a case of angeio-neurotic œdema which he had under observation from 1886 to 1902, in which year the patient died of acute œdema of the larynx. She had suffered from the disease since childhood, and her father had presented similar symptoms and died in the same manner. Dr. Griffith gives a review of the literature, and while accepting the nervous origin of the disease, holds that it has some analogies to the œdema produced by certain drugs and by the poison of rheumatism, gout, and even some cardiac and hepatic conditions.

**Purpura Hæmorrhagica.**—A case of Henoch's disease was described.[16] The pathology was also discussed, the theory being that slighter cases are due to a vasomotor disturbance leading to dilatation, stasis and rupture in the smaller vessels. In fatal cases endarteritic changes have been found. Relapses are frequent and the prognosis should be guarded. Lang Gordon[17] describes an exceptionally severe case of articular swellings combined with hæmorrhages, rashes of a purpuric, urticarial and erythematous nature, colic and pyrexia. The patient, a boy of 14, was continuously ill for 90 days. Towards the end a systolic mitral bruit developed, and shortly afterwards a mild attack of chorea occurred. Jacobson[18] describes a case of purpura in a woman aged 37. She appeared to have appendicitis, but at the operation only interstitial hæmorrhages in the vermiform appendix were found. Subsequently purpura and hæmorrhages from the respiratory tract occurred. There were no arthritic or gastro-intestinal symptoms, and no family or personal history of hæmorrhagic disease. Supra-renal extract had a markedly beneficial effect. Patterson[19] reports a case in which the common femoral and subsequently the external iliac were ligatured in the hope of controlling a large subcutaneous hæmorrhage in a "bleeder." The patient, however, died. Adrenalin, calcium, chloride and subcutaneous injections of gelatine were ineffectual. The patient had had a previous operation for osteomyelitis successfully performed in an apparently dormant period of the disease. The pathology is discussed, and Grüber's view that the delayed clotting is due to the prolonged retention in the leucocytes of a nucleo-proteid essential to the formation of fibrin is mentioned as a possible indication for treatment in the future.

[1] Lancet, Jan. 31, 1903, p. 288. [2] Brit. Med. Jour., Mar. 7, 1903, p. 549. [3] Ibid., June 14, 1902, p. 1461. [4] Ibid., Jan. 31, 1903, p. 243. [5] Amer. Jour. Med. Sci., Feb. 1903, p. 273. [6] Brit. Med. Jour., Jan. 31, 1903, p. 237. [7] Amer. Jour. Med. Sci., Mar. 1903, p. 413. [8] Brit. Med. Jour., Jan. 3, 1903, p. 19. [9] Ibid., Feb. 14, 1903, p. 367. [10] Ibid., Supplement, Feb. 7, 1903. p. 15. [11] Ibid., Mar. 7, 1903, p. 543. [12] Lancet, Feb. 7, 1903, p. 388. [13] Med. Rec., Feb. 2, 1903, p. 226. [14] Lancet, Jan. 24, 1903, p. 228. [15] Brit. Med. Jour., June 14, 1902, p. 1470. [16] Med. Press, Jan. 29, 1902, p. 117. [17] Lancet, Feb. 14, 1903, p. 433. [18] Med. Rec., Feb. 7, 1903 p. 207. [19] Scot. Med. and Surg. Journ., Feb. 1903, p. 136.

